# Behavioural influences of critical care physicians and nurses intention to implement preventive evidence-based measures towards central line associated blood stream infections (CLABSIS)

**DOI:** 10.1186/2197-425X-3-S1-A890

**Published:** 2015-10-01

**Authors:** K Iliopoulou, J Anderson, T Day

**Affiliations:** Midwifery and Nursing Faculty, King's College Hospital, London, United Kingdom

## Introduction

Although CLABSIs within Intensive Care Units (ICUs) can be prevented through the implementation of evidence-based measures, overall compliance is consistently suboptimal amongst health care workers (HCWs). Furthermore, little is known about factors that motivate critical care physicians and nurses to apply evidence-based measures towards CLABSIs prevention.

## Objectives

The study aimed to examine the relationship between attitudes, social norms, self-efficacy and underlying beliefs and critical care physicians' and nurses' intention to implement evidence-based practice towards CLABSIs prevention.

## Methods

A questionnaire based on attitude-social norms-self efficacy model was administered to a convenience sample of critical care HCWs (n = 77 physicians, n = 100 nurses; response rate 81%). Eighty-eight nurses and fifty-six physicians completed a 51-item and 56-item questionnaire respectively, working in four ICUs located in two universities and one urban hospital in Greece. Data were collected on physicians and nurses self-reported compliance, attitude, social norms and self-efficacy.

## Results

HCWs reported high compliance while nurses reported higher compliance than physicians (*p = 0.027*).Physicians and nurses reported moderate confidence in their ability (self-efficacy) to implement the preventive measures towards CLABSIs. Lack of supplies was reported as a barrier by both groups while low standards of care and lack of sufficient time were reported as barriers only by nurses. Ineffective dissemination of infection control policies and inadequate in-service education were reported as barriers by physicians. HCWs reported a positive attitude towards implementation of CLABSIs preventive measures. A higher perceived pressure from colleagues to perform the above behavior was reported by physicians, in comparison to nurses (*p*< 0.001). Moreover, medical/nurse directors appeared not to be perceived as role models in regards to CLABSIs prevention. Bivariate analysis of the relationship between self-reported compliance and model's variables revealed that there was a moderate relationship between the general attitude towards the behavior and self-reported compliance (*r* = 0.34, p = 0.01, *r* = 0.33, p < 0.001, respectively). Linear regression analysis revealed that a positive attitude towards CLABSIs prevention was independently associated with intention to implement the desired behavior for HCWs (b=.185; *p* = 0.011 for physicians, b=.246; *p* = 0.005 for nurses).

## Conclusions

Findings suggest that interventions to increase compliance with CLABSIs preventative measures in critical care settings need to target antecedent attitudes and beliefs that are related to behavioral compliance. Further research should explore the influence of organizational factors such as leadership on intention to apply these.
